# Integrating multiple regulations on enzyme activity: the case of phospho*enol*pyruvate carboxykinases

**DOI:** 10.1093/aobpla/plad053

**Published:** 2023-08-02

**Authors:** Bruno E Rojas, Alberto A Iglesias

**Affiliations:** Instituto de Agrobiotecnología del Litoral, UNL, CONICET, FBCB, Santa Fe, Argentina; Instituto de Agrobiotecnología del Litoral, UNL, CONICET, FBCB, Santa Fe, Argentina

**Keywords:** Allosteric regulation, carbon metabolism, enzyme regulation, gluconeogenesis, metabolic control, photosynthesis, phosphoenolpyruvate carboxykinases, post-translational modification

## Abstract

Abstract. Data on protein post-translational modifications (PTMs) increased exponentially in the last years due to the refinement of mass spectrometry techniques and the development of databases to store and share datasets. Nevertheless, these data per se do not create comprehensive biochemical knowledge. Complementary studies on protein biochemistry are necessary to fully understand the function of these PTMs at the molecular level and beyond, for example, designing rational metabolic engineering strategies to improve crops. Phospho*enol*pyruvate carboxykinases (PEPCKs) are critical enzymes for plant metabolism with diverse roles in plant development and growth. Multiple lines of evidence showed the complex regulation of PEPCKs, including PTMs. Herein, we present PEPCKs as an example of the integration of combined mechanisms modulating enzyme activity and metabolic pathways. PEPCK studies strongly advanced after the production of the recombinant enzyme and the establishment of standardized biochemical assays. Finally, we discuss emerging open questions for future research and the challenges in integrating all available data into functional biochemical models.

## Teaser

Plant biochemistry studies enzymes and metabolic pathways regulation in plants, crucial for devising effective crop improvement strategies. The advent of advanced mass spectrometry techniques, protocols, and specialized databases has facilitated access to valuable post-translational modification data on plant proteins. Phospho*enol* pyruvate carboxykinases (PEPCKs) have diverse roles in plant physiology, including C_4_ and crassulacean acid metabolism (CAM) photosynthesis, carbon fixation in algae, gluconeogenesis during seed germination and fruit maturation, stress response, and amino and organic acids metabolisms. PEPCKs exemplify the complex regulation of metabolic enzymes, encompassing allosteric effectors (metabolites and dipeptides), proteolysis, and phosphorylation, which are explained in detail in this review. Further research is needed to deepen our understanding of this intricate regulation and advance metabolic engineering and synthetic biology in plants.

## Introduction

**Figure 1.**
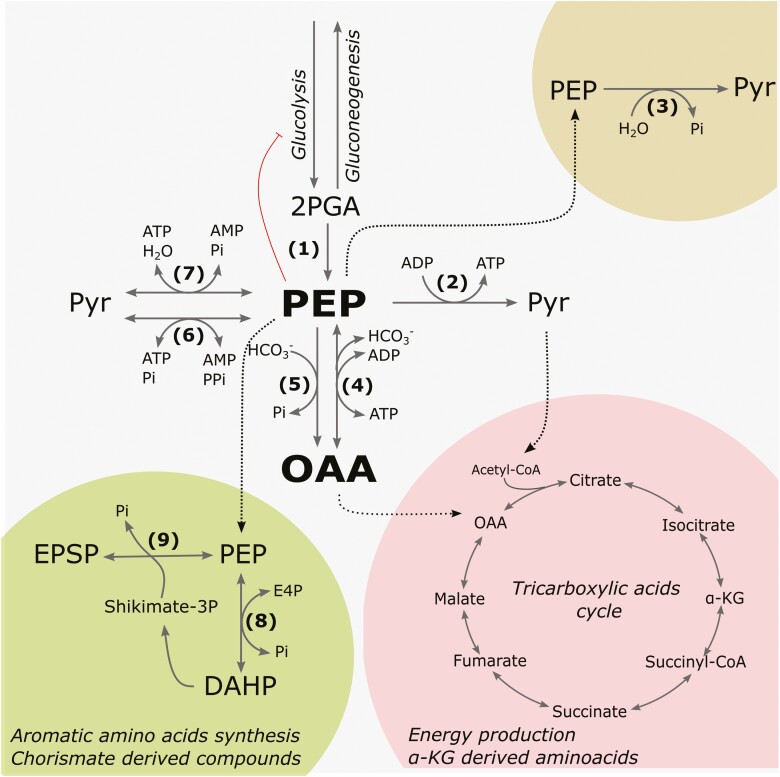
**General overview of the metabolic pathways and enzymes that converge on PEP in plants.** (1) Enolase (EC 4.2.1.11), (2) Pyr kinase (EC 2.7.1.40), (3) PEP phosphatase (3.1.3.60), (4) ATP-dependent PEPCK (EC 4.1.1.49), (5) PEP carboxylase (EC 4.1.1.31), (6) Pyr PPi dikinase (EC 2.7.9.1), (7) PEP synthase (EC 2.7.9.2), (8) DAHP synthase (EC 2.5.1.54), (9) 3-phosphoshikimate 1-carboxyviniltransferase (EC 2.5.1.19). DAHP, 3-deoxy-7-phosphoheptunolate; E4P, erythrose-4-phosphate, ESPS, 5-5-*enol*pyruvylshikimate 3-phosphate.

Plant biochemistry focuses on understanding the kinetics, structure and regulatory mechanisms that govern enzymes and metabolic pathways in plants (Hartman *et al.,* 2023). This historical approach is essential to understand biological processes in detail and is the foundation of rational strategies in metabolic engineering and synthetic biology for crop improvement (Wurtzel *et al.* 2019). Over the past two decades, the development of mass spectrometry techniques coupled with specialized databases for data storage and sharing has resulted in an explosion of plant protein post-translational modifications (PTMs; Smith and Kelleher 2013; Aebersold *et al.* 2018; Willems *et al.* 2019). Furthermore, the refinement of mass spectrometry protocols allowed the detection of low-stoichiometry PTMs, which were previously considered unimportant but are now being revisited with renewed interest (Prus *et al.* 2019). Having plenty of data/information does not translate into meaningful and practical knowledge/understanding (Ackoff 1999; Dammann 2019). A combined array of genetic and biochemical techniques is necessary to truly comprehend the function of PTMs and their integration with our knowledge of enzyme regulation (Stitt and Gibon 2014). This integrated approach is exemplified by the study of phospho*enol*pyruvate carboxykinases (PEPCKs), which are critical in central metabolism and physiological processes (Fig. 1). The biological relevance of PEPCK is associated with its complex regulation, recently evidenced by combining genetic, biochemical and systems biology approaches. Finally, we discuss open questions, which determine future lines of research.

### Plant PEPCK characteristics

PEPCKs catalyse the decarboxylation of oxaloacetate (OAA) to PEP through an *enol* intermediate that is phosphorylated using adenosine triphosphate (ATP) (EC 4.1.1.49), guanosine triphosphate (GTP) (EC 4.1.1.32) or inorganic pyrophosphate (PPi) (EC 4.1.1.38), following a nucleophilic substitution of the S_N_2 type (Matte *et al.* 1997; Holyoak *et al.* 2006; Johnson *et al.* 2016). Although the reaction is fully reversible *in vitro*, at least in plants, it mainly courses into the OAA decarboxylation direction *in vivo*. PEPCKs need two metal ions for optimal activity, one that complexes with the nucleotide substrate and the other that acts as a cofactor and might bind the enzyme in the absence of the substrate (Lee *et al.* 1981; Matte *et al.* 1997; Holyoak *et al.* 2006; Rojas *et al.* 2019). The nucleotide is activated by Mg^2+^ and Mn^2+^, while a transition metal like Mn^2+^, Co^2+^ or Ca^2+^ acts as the cofactor. The cofactor promotes the decarboxylation of OAA through the formation of an intermediary complex that stabilizes the enolate ion during catalysis (O´Leary 1992; Matte *et al.* 1997; Johnson *et al.* 2016).

ATP-dependent PEPCKs were found in bacteria, yeasts and plants, while those using GTP are present in mammals, archaea and a small group of bacteria (Fukuda *et al.* 2004; Aich and Delbaere 2007; Chiba *et al.* 2015; Rojas *et al.* 2019). The distribution of PPi-dependent PEPCKs is less clear; this activity was discovered in crude extracts of *Propionibacterium shermanii*, and the coding gene was later identified in *Entamoeba histolytica* (Siu *et al.* 1961; Chiba *et al.* 2015). PEPCKs differ in their quaternary structure, being the GTP-dependent monomeric (Matte *et al.* 1997), the PPi-dependent homodimeric (Chiba *et al.* 2019) and the ATP-dependent homomultimeric, conformed either by four or six subunits (Burnell 1986; Walker and Leegood 1995; Martín *et al.* 2011; Rojas *et al.* 2019; Toressi *et al.* 2023).

The evolutionary trajectory of PEPCKs is a matter of controversy. Some authors have suggested that there is no homology between ATP- and GTP-dependent PEPCKs, but their primary sequence has similar motifs for substrate and metal binding (Matte *et al.* 1997; Fukuda *et al.* 2004; Aich and Delbaere 2007). PPi-dependent PEPCKs lack the catalytic domains described for the ATP- and GTP-dependent PEPCKs, so the evolutionary distance might be higher (Chiba *et al.* 2015). On the contrary, other authors have concluded that all PEPCK forms likely arose from a common ancestor (Walker and Chen 2002, 2021). Supporting this view, the tri-dimensional protein structures are similar, reinforcing that different PEPCKs are homologous (Chiba *et al.* 2019).

ATP-dependent PEPCKs have cytosolic localization in higher plants (Reiskind and Bowes 1991; Ito *et al.* 2011; Tsiatsiani *et al.* 2013). Other photosynthetic organisms, like the diatom *Skeletonema costatum* and the algae *Laminaria setchellii* contain a chloroplastic PEPCK (Cabello-Pasini *et al.* 2001). The characteristics of the reaction catalysed by PEPCKs attach them to critical energetic nodes in metabolism, independently of the organism. Let’s analyse the particularities of the physiological role of PEPCKs in plants.

### Physiological roles

#### Decarboxylation of C_4_ acids in C_4_ and crassulacean acid metabolism photosynthesis.

In C_3_ photosynthesis, ribulose-1,5-bisphosphate carboxylase-oxygenase (RuBisCO) mediates CO_2_ fixation into ribulose-1,5-bisphosphate, to produce two molecules of 3-phosphoglycerate (3PGA, three-carbon). This reaction is the initial step of the Calvin–Benson–Bassham cycle (CBBc), a chloroplast carbon cycle that sustains all life on earth (Sage 2004; Rojas *et al.* 2021a). The oxygenase activity of RuBisCO can fix O_2_ instead of CO_2_, generating 3PGA and 2-phosphoglycolate. As this latter two-carbon intermediate is toxic to the plant, it has to be recycled through photorespiration, which leads to energy, carbon and nitrogen losses, decreasing plant productivity (Portis 2001; Trípodi *et al.* 2021; Rojas *et al.* 2021a).

**Figure 2.**
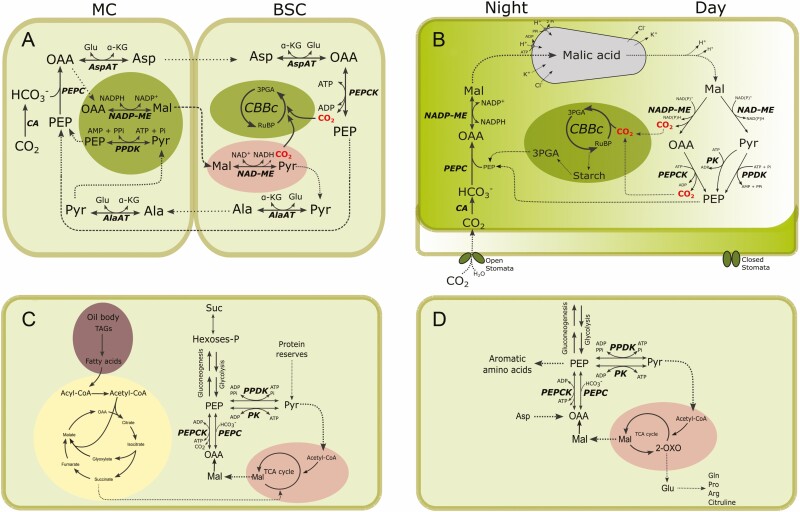
**Summary of the physiological roles of PEPCKs.** (A) The CO_2_-concentrating mechanisms operating in C_4_ plants. PEPCKs decarboxylate, in the cytosol, the OAA resulting from Asp decarboxylation. Then, the resulting PEP returns to the MC to restart the C_4_ cycle. MC; mesophyll cells, BSC; bundle sheath cells. (B) Mal cycling in CAM plants. CAM plants open their stomata during cold humid nights to incorporate CO_2_, reducing H_2_O loss. The CO_2_ is incorporated into Mal, which accumulates inside the vacuole. During hot dry days, CAM plants keep their stomata closed, and Mal is released from the vacuole to be decarboxylated and the resulting CO_2_ fixed into the CBBc. (C) Gluconeogenesis during oilseeds germination. TAGs are degraded in a process distributed between different pathways and organelles. PEPCKs and PPDKs represent the first step of the gluconeogenic pathway. (D) Amino and organic acid metabolism. PEPCKs localize at a metabolic node between the amino acids derived from PEP and those derived from intermediates of the TCA cycle. AspAT, Asp aminotransferase; AlaAT, Ala aminotransferase; CA, carbonic anhydrase; NAD-ME, NAD-dependent malic enzyme; NADP-ME, NADP-dependent malic enzyme; PEPC, PEP carboxylase; PEPCK, ATP-dependent phosphoenolpyruvate carboxykinase; PK, Pyr kinase; PPDK, Pyr PPi dikinase.

C_4_ and crassulacean acid metabolism (CAM) photosynthesis evolved from C_3_ ancestors to mitigate the detrimental effects of photorespiration and water loss by transpiration, respectively (Sage 2004; John *et al.* 2014; Trípodi *et al.* 2021). The different lineages of C_4_ plants developed the Kranz anatomy, differentiation between mesophyll (MC) and bundle sheath cells (BSC), as well as CO_2_-concentrating mechanisms. The latter include the initial fixation of CO_2_ by phospho*enol*pyruvate carboxylase (PEPC) in mesophyll cells, transport of C_4_ acids to BSC and decarboxylation near RuBisCO, enabling efficient carbon fixation via the CBBc. Based on the primary decarboxylase employed in the process, plant lineages were classified as NAD-dependent malic enzyme (NAD-ME, EC 1.1.1.38-39), NADP-dependent malic enzyme (NADP-ME, EC 1.1.1.40) and PEPCK subtypes (Hatch *et al.* 1975; John *et al.* 2014). In the PEPCK lineage, malate (Mal) and Asp are transported to the BSC. Within these cells, Asp is decarboxylated by the consecutive action of Asp aminotransferase (EC 2.6.1.1) and PEPCK in the cytosol, while Mal decarboxylation courses through mitochondrial NAD-ME (Maier *et al.* 2011) (Fig. 2A).

Despite the above-mentioned classification, decarboxylation pathways are flexible and might operate simultaneously in diverse species (Furbank 2011; Maier *et al.* 2011; Wang *et al.* 2014). In maize (NADP-ME subtype), carbon is primarily fixed as Mal, but significant pools (~25% of the fixed carbon) and rapid labelling of Asp have also been observed (Hatch 1971; Meister *et al.* 1996; Wingler *et al.* 1999; Majeran *et al.* 2010; Pick *et al.* 2011; Muhaidat and McKown 2013; Weissmann *et al.* 2015; Arrivault *et al.* 2017). Furthermore, PEPCK is expressed in the BSC of the NADP-ME subtype and supports Asp-dependent photosynthesis in isolated BSC (Chapman *et al.* 1980; Chapman and Hatch 1981; Walker *et al.* 1997; Wingler *et al.* 1999; Majeran *et al.* 2010; Pick *et al.* 2011; Washburn *et al.* 2021). It has been suggested that Asp decarboxylation varies with developmental and environmental conditions (Chapman and Hatch 1981; Furbank 2011). Also, in sugarcane (classified as NADP-ME subtype), PEPCK activity is induced during sugar accumulation, drought and shade stress (Sales *et al.* 2018; Cacefo *et al.* 2019; Marquardt *et al.* 2021). However, this is controversial as other authors have shown that sugarcane does not have substantial PEPCK (Walker *et al.* 1997; Sage *et al.* 2013).

In CAM photosynthesis, atmospheric CO_2_ fixation also produces a C_4_ acid. However, in this case, the initial carbon assimilation by PEPC and the following incorporation into the CBBc are temporary. CAM plants have a high water-use efficiency, which allows them to inhabit desertic environments. They open their stomata to capture CO_2_ during cold and humid nights and keep them closed on hot and dry days, reducing transpiration to a minimal level (Trípodi *et al.* 2021). During the night, CO_2_ is fixed by the enzymatic tandem carbonic anhydrase (EC 4.2.1.1), PEPC and Mal dehydrogenase, and the product Mal is then stored in the vacuole. During the day, the hydroxy acid undergoes decarboxylation by NAD(P)-ME or PEPCK, releasing CO_2_ near RuBisCO to feed the CBBc (Dittrich *et al.*, 1973) (Fig. 2A). CAM plants were classified into two groups, one that decarboxylates Mal via NAD(P)-ME (with undetectable PEPCK) and another that uses PEPCK (having low NAD(P)-ME activity) (Dittrich *et al.* 1973; Holtum and Osmond 1981; Borland *et al.* 1998) (Fig. 2B). However, it is worth noting that this classification is not rigid, and there is evidence of multiple decarboxylase activities (similar to what happens to C_4_ plants) present in CAM plants, at similar levels (Black *et al.* 1996; Peckmann *et al.* 2012; Mukundan *et al.* 2023). This would give CAM photosynthesis increased efficiency and plasticity under different physiological and environmental conditions. In *Mesembryanthemum crystallinum*, a species that performs C_3_ photosynthesis but can switch to CAM in response to salinity or water-deficit stress, the *PEPCK* gene has a stress-inducible expression that peaks at dawn, indicating a potential role of PEPCK in CAM induction (Lim *et al.* 2019).

#### Carbon fixation in algae.

Giordano *et al*. (2005) reviewed the evidence for the existence of a C_4_-like metabolism in algae, although the collected evidence is inconclusive. In the marine alga *Udotea flabellum*, which exhibits C_4_-like photosynthesis without cellular compartmentalization, the carboxylase activity of PEPCK was found to be equivalent to that of RuBisCO (Reiskind and Bowes 1991). These authors also reported that 3-mercaptopiconilic acid (3-MPA), a specific inhibitor of PEPCK (Leegood and Ap Rees 1978*a*, *b*), reduced photosynthesis by 70%, highlighting the enzyme’s role in carbon fixation in this organism (Reiskind and Bowes 1991). In the diatom *Thalassiosiva weissflogii*, which has a C_4_-like metabolism, PEPCK may also decarboxylate C_4_ acids (Reinfelder *et al.* 2004). In the diatoms *S. costatum* and *L. setchellii*, the chloroplastic localization of PEPCK coincides with its role as a decarboxylase that delivers CO_2_ near RuBisCO (Cabello-Pasini *et al.* 2001). In *Phaeodactylum tricornutum*, PEPCK localizes in the mitochondria, and knockdown of the gene encoding this enzyme reduces growth and photosynthesis while increasing TAG accumulation under nitrogen-limiting conditions (Yang *et al.* 2016). The PEPCK role in algae not only could be related to carbon fixation, but also to gluconeogenesis and organic acids and nitrogen metabolisms (Toressi *et al.* 2023).

#### Gluconeogenesis in oleaginous seeds.

The work of Leegood and Ap Rees (1978*a*, *b*) provided important supportive data for the role of PEPCK in plant gluconeogenesis. These authors studied PEPCK activity in cucumber cotyledons and purified the enzyme from this source. They also demonstrated that treatments with 3-MPA halted the gluconeogenic flux in this system (Leegood and Ap Rees 1978*a*, *b*). The accumulation of PEPCK coincided with those of other gluconeogenic enzymes, such as isocitrate lyase and Mal synthase, in the cotyledons of *Cucurbita pepo* and *Cucumis sativus* (Kim and Smith 1994), *Arabidopsis thaliana* (Rylott *et al.* 2001) and *Ricinus communis* (Martín *et al.* 2007), a few days after the imbibition of seeds. Later, genetic studies in *A. thaliana* furthered the understanding of the physiological functions of PEPCKs. Arabidopsis has two genes coding for ATP-dependent PEPCKs: *PEPCK1* (At4g37870) and *PEPCK2* (At5g65690). The *PEPCK1* gene is predominantly expressed during seed germination, while *PEPCK2* expression is less abundant and restricted to specific plant tissues (Malone *et al.* 2007; Rojas *et al.* 2021b).

The study of knock-out mutants and silenced lines in *PEPCK1* provided further insight into the critical involvement of PEPCK in plant gluconeogenesis (Rylott *et al.* 2003; Penfield *et al.* 2004). These mutants exhibited growth impairment when cultivated on basal media without an external carbon source, but their growth was normal when the media was supplemented with sucrose (Rylott *et al.* 2003; Penfield *et al.* 2004). Once the seedlings developed their photosynthetic apparatus, their growth was similar to that of wild-type (WT) plants. Additionally, these mutants did not alter the catabolism rate of reserve lipids, but showed reduced levels of soluble sugars. However, under suboptimal illumination or dark conditions, their growth was further compromised, resulting in reduced hypocotyl elongation compared to WT plants. This reduction in hypocotyl elongation was also observed when the endosperm, the tissue where oleaginous plants store reserve lipids, was eliminated. Hence, these phenotypes could be attributed to a decrease in the sucrose supply received by the embryo from the endosperm (Penfield *et al.* 2004).

As *pepck1* mutants can develop their photosynthetic apparatus and mature into adult plants, the step catalysed by PEPCK, although critical, is not the only path through which carbon could flow to gluconeogenesis (Rylott *et al.* 2003; Penfield *et al.* 2004). Eastmond *et al.* (2015) discovered that Arabidopsis employs two pathways to channel carbon from reserves to gluconeogenesis: one via PEPCK, which channels carbon from lipids degradation, and the other via PPDK, which channels carbon from protein reserves (Fig. 2C). In Arabidopsis, *PPDK* has an expression pattern like *PEPCK1*, peaking approximately 2 days after imbibition. Seedling establishment is impaired in *ppdk* mutants, and the double-mutant *ppdk/pepck1* displays a severe starvation response (Eastmond *et al.* 2015). This two-enzyme model for carbon channelling during Arabidopsis seeds germination was subsequently confirmed by Henninger *et al.* (2022).

The contribution of PEPCK and PPDK to gluconeogenesis during seed germination may vary across plant species. For instance, germinating cucumber cotyledons exhibit abundant PEPCK activity but undetectable levels of PPDK (Walker *et al.* 2021). Therefore, it is important to investigate the prevalence of both enzymes in gluconeogenesis during germination across different plants, considering factors such as species, tissue type, developmental stage and environmental conditions like nitrogen supply (Walker *et al.* 2021). Moreover, distinct signalling cascades may regulate the involvement of each enzyme in gluconeogenesis. For example, in Arabidopsis seedling establishment, the SnRK1 kinase serves as a critical regulator of reserve mobilization. SnRK1 phosphorylates the transcription factor BASIC LEUCINE ZIPPER63 which binds and activates the *PPDK* promoter. Notably, SnRK1 does not appear to regulate *PEPCK1* (Henninger *et al*. 2022), suggesting that gluconeogenesis may be controlled through complex interactions of multiple signalling pathways.

#### Gluconeogenesis during fruit maturation.

Studies have demonstrated that gluconeogenesis occurs in several fruits (Sweetman *et al.* 2009; Walker *et al.* 2016; extensively reviewed in Walker *et al.* 2021) with PEPCK (rather than PPDK) being the primary pathway utilized, except possibly in tomato (Famiani *et al.* 2014, 2016). Gluconeogenesis from Mal and citrate can occur in certain fruits even when there is no net breakdown of these organic acids. It seems that gluconeogenesis in fruits is associated with a transient release of Mal and/or citrate from the vacuole (Walker *et al.* 2021). In tomato fruits, silencing the *PEPCK* gene by interfering RNA led to a decrease in sugar concentration and increased levels of Mal (Osorio *et al.* 2013; Huang *et al.* 2015*a*). Conversely, overexpressing the same gene using a constitutive (*35SCAMV*) or fruit-specific promoters (fruit-ripening-specific *E8* promoter; Huang *et al.* 2015*b*) resulted in the opposite phenotype, along with faster germination and seedling growth.

#### Stress response.

In Arabidopsis, *PEPCK1* is expressed in biotic stress-response structures like hydathodes, trichomes and guard cells (Chen *et al.* 2000, 2002; Penfield *et al.* 2012). In *Capsicum annum*, a *PEPCK* gene was isolated from a cDNA library constructed from leaves infected with an avirulent *Xanthomonas* strain (Choi *et al.* 2015). Following infection, PEPCK expression and activity increased, suggesting its involvement in biotic stress response. Additionally, silencing this gene in pepper plants rendered them more susceptible to infection, while overexpression increased resistance (Choi *et al.* 2015). The authors of this work also observed that drought, cold and stress-related hormones (such as salicylic acid and abscisic acid, ethylene and methyl-jasmonate) triggered the induction of the *PEPCK* gene.

Arabidopsis *pepck1* mutants are susceptible to drought due to stomatal malfunction resulting in increased transpiration (Daloso *et al.* 2017). Mal metabolism in guard cells plays a crucial role in the opening and closure of stomata (Daloso *et al.* 2017; Robaina-Estévez *et al.* 2017). In *pepck1* mutants, anomalies in Mal metabolism within these cells may explain the malfunctioning of this mechanism, as ABA signalling is not affected (Penfield *et al.* 2012). Furthermore, the induction of the *PEPCK* gene has been observed in tomato plants exposed to saline stress (Saito *et al.* 2008) and in *Brassica napus* leaves subjected to cold stress (Saez-Vasquez *et al.* 1995). In sugarcane, PEPCK activity is induced during drought stress and shade conditions (Sales *et al.* 2018; Cacefo *et al.* 2019).

#### Cataplerotic reactions, amino acid metabolism and cytosolic pH regulation.

The removal of intermediates from the tricarboxylic acid (TCA) cycle is crucial to prevent their accumulation in specific metabolic situations. These enzymatic steps, known as cataplerotic reactions, are critical to cellular homeostasis (Owen *et al.* 2002). In the case of PEPCK, it converts OAA into PEP, which can undergo gluconeogenesis or generate Pyr (Leegood and Walker 2003) (Fig. 2D). In plants, cataplerotic reactions are important during certain physiological conditions, such as castor oil seeds germination (Stewart and Beevers 1967), amino acid respiration in *Pisum sativum* (Larson and Beevers 1965) or the use of glutamate as a respiratory substrate in sugar beet phloem (Lohaus *et al.* 1994). However, the precise role of PEPCK in these plant processes requires further investigation.

The PEPCK reaction is localized between the OAA/Asp family of amino acids (Asn, Lys, Thr, Met and Ile), those derived from PEP (Phe, Tyr and Trp), and Ala derived from Pyr (Lea *et al.* 2001) (Fig. 2D). In grape seeds, PEPCK activity is induced by nitrogenous compounds (Asp, NH_4_^+^ and Gln), suggesting a potential role in their metabolism and the regulation of cytosolic pH through Mal formation and dissimilation (Walker *et al.* 1999; Lea *et al.* 2001; Chen *et al.* 2004; Delgado-Alvarado *et al.* 2007). Decarboxylases of C_4_ acids are localized in the mid-vein of Arabidopsis, and their mutation affects the abundance of amino acids derived from Pyr and PEP. By feeding the xylem stream of the Arabidopsis *pepck1* mutant with ^14^C-labelled bicarbonate and Mal, it was observed that the levels of Ala (derived from PEP) were reduced, while Asp (produced from OAA) increased compared to WT plants (Brown *et al.* 2010).

### Regulatory mechanisms

#### Allosteric regulation.

Over the years, and under certain assay conditions, several metabolites have been shown to affect PEPCK activity *in vitro*. The enzymes from *Urochloa panicoides*, *Chloris gayana* and *Panicum maximum* (C_4_ plants) are inhibited by the glycolytic intermediates 3PGA, Fru6P, Fru1,6bisP and DHAP under certain conditions (Hatch and Mau 1977; Burnell 1986). For the maize (also performing C_4_ photosynthesis) PEPCK, sensitivity to 3PGA inhibition is only evident in the enzyme’s N-terminal proteolyzed form (Furumoto *et al.* 1999). A detailed study on the allosteric regulation of a short version of the enzyme from *Ananas comosus* (a CAM species) revealed differential regulation between its carboxylase and decarboxylase activities (Martín *et al.* 2011). The decarboxylase activity was inhibited by Fru2,6bisP, 3PGA, Asp and Pro, while succinate activated it. The carboxylase activity was inhibited by Fru6P, Fru1,6bisP, 3PGA, citrate, Mal and UDPGlc (Martín *et al.* 2011). However, the authors of this study were unable to purify the full-length form of the enzyme to make a comparative assessment of differences in allosteric regulation. *Chlamydomonas reinhardtii* has two PEPCKs; a full-length *Chlre*PEPCK1 and a shorter *Chlre*PEPCK2 that lacks 55 amino acids at the N-terminus. *Chlre*PEPCK1 and *Chlre*PEPCK2 carboxylase activity is inhibited by citrate and phenylalanine. *Chlre*PEPCK2 carboxylase activity is also inhibited by glutamine. The decarboxylase activity of *Chlre*PEPCK1 is activated by phenylalanine and malate, while *Chlre*PEPCK2 decarboxylase activity does not show any effect by these metabolites and is inhibited by glutamine (Toressi *et al.* 2023).

It is important to note that PEPCK activity was typically assayed using relatively high concentrations of Mn^2+^ (0.5–5 mM), which allow for maximal activity. However, the concentration of this metal in plant cells remains within the micromolar range (Quiquampoix *et al.* 1993; Pittman 2005). Improvements to the *in vivo* assay of PEPCK allowed the enzyme’s activity to be measured at more physiological concentrations of metal ions and so, physiologically relevant conclusions could be obtained from the *in vitro* assays (Chen *et al*. 2002; Rojas *et al*. 2019). In the case of Arabidopsis (a C_3_ plant), there are differences in the regulation of *Ath*PEPCK1 and *Ath*PEPCK2 (Rojas *et al.* 2019). Glc6P, Fru6P and Glc1P inhibit *Ath*PEPCK1 but not *Ath*PEPCK2, while Fru1,6bisP inhibits both enzymes. Glc6P is the primary inhibitor of *Ath*PEPCK1, followed by Glc1P, Fru6P and Fru1,6bisP. Mal activates *Ath*PEPCK1 but not *Ath*PEPCK2. The regulation by Mal may be critical in stimulating the flux of carbon released through lipid degradation into gluconeogenesis during seed germination. Once the photosynthetic apparatus develops, increased levels of triose- and hexose-phosphates may inhibit PEPCK, thereby inhibiting gluconeogenesis. *Ath*PEPCK1 is also inhibited by shikimate, a precursor of aromatic amino acids and defence compounds, thus regulating the synthesis of PEP, the initial substrate of the shikimate pathway (Rojas *et al.* 2019). The regulation of PEPCK by Mal and Glc6P is opposite to that of PEPC, which may be an important mode of regulation for two enzymes catalysing opposite reactions and present in the plant cytosol simultaneously. This regulation could be key in preventing a futile carboxylation/decarboxylation cycle that would deplete cytosolic ATP (Leegood and Walker 2003; Martín *et al*. 2011; Rojas *et al*. 2019).

#### Regulation by dipeptides.

Identifying novel protein–metabolite interactions is crucial for discovering new regulatory elements in plant metabolism. For this, PROMIS (*PROtein Metabolite Interactions using Size separation*) proved to be a valuable tool for mining novel small molecule regulators (Veyel *et al.* 2017, 2018). *Ath*PEPCK1 co-elutes with a series of hydrophobic/polar dipeptides: Ile-Gln, Ala-Ile, Phe-Gln, Leu-Thr, Ser-Tyr, and Ser-Val. Enzymatic assays demonstrated that these dipeptides inhibit recombinant *Ath*PEPCK1 with *I*_0.5_ values ranging from 52 to 828 µM. They can also inhibit PEPCK activity in crude extracts, and this inhibition is not observed with the individual amino acids (Moreno *et al.* 2021). The inhibition of *Ath*PEPCK1 by dipeptides is more potent than that of sugar phosphates (Rojas *et al.* 2019; Moreno *et al.* 2021). The origin and roles of these regulatory dipeptides are still not fully understood. Additionally, the dipeptide’s *in vivo* concentration range remains unknown, which is important for determining if they can affect PEPCK activity *in vivo*. It is possible that they participate in signalling during abiotic stress in Arabidopsis (Doppler *et al.* 2019; Thirumalaikumar *et al.* 2021). Moreover, dipeptides have been detected in Arabidopsis root exudates, downstream of the MPK3 and MPK6 signalling cascade, suggesting their involvement in plant–microbe and plant–plant communication systems (Strehmel *et al.* 2017).

Arabidopsis mutants in components of the autophagy pathway, including (*atg18* and *nbr1-2*) simple mutants as well as [*atg4(4a/4b)* and *nbr1-2/atg5*] double mutants exhibited reduced dipeptides levels upon heat stress when compared to the WT (Thirumalaikumar *et al.* 2021). Autophagy plays a critical role in macromolecule recycling (Kenny *et al.* 1976) and seedling germination (Avin-Wittenberg *et al.* 2015). Arabidopsis mutants in genes involved in the autophagic response are sensitive to carbon and nitrogen starvation, which can be alleviated by supplying sucrose to the seedlings (Bassham 2009). Also, these mutants exhibit decreased levels of free amino acids and increased protein content, indicating impaired mobilization of reserve proteins and lipids (Avin-Wittenberg *et al.* 2015). Notably, one of the increased proteins was CRUCIFERIN3, a major reserve protein in Arabidopsis seeds (Herman and Larkins 1999; Wan *et al.* 2007).

It would be intriguing to explore whether the inhibition of *Ath*PEPCK1 by H-P dipeptides regulates the channelling of carbon released through protein degradation during seed germination via PPDK, which does not interact with H-P dipeptides (Veyel *et al.* 2018). Arabidopsis seeds overexpressing the sunflower WRKY10 transcription factor exhibited increased gluconeogenesis, enhanced lipid utilization, reduced protein consumption and a higher flux through PEPCK during germination (Raineri *et al.* 2016).

#### Proteolytic regulation.

Plant PEPCKs undergo proteolysis at the N-terminus *in vivo*. The proteolysis is affected by the pH (diminishing at pH 9–10), cannot be avoided by protease inhibitors and does not alter the quaternary structure of the enzyme (Walker and Leegood 1995; Walker *et al.* 1995; Rojas *et al.* (2021b)). In Arabidopsis, *Ath*PEPCK1 is a target of the cysteine-protease METACASPASE9 (*Ath*MC9) (Tsiatsiani *et al.* 2013). *Ath*MC9 is found in the nucleus, cytosol and apoplast (Vercammen *et al.* 2006; Kwon and Hwang 2013; Tsiatsiani *et al.* 2013), and participates in regulating cellular death in various physiological contexts, such as the immune response (Kim *et al.* 2013; Shen *et al.* 2019) and vascular tissue development (Escamez *et al.* 2016, 2019). Proteolysis of the N-terminal domain of PEPCK appears to activate the enzyme, as crude extracts from the Arabidopsis *mc9* mutant exhibit decreased enzyme activity, while *35S:MC9* overexpressing lines show increased PEPCK activity (Tsiatsiani *et al.* 2013). The level of *Ath*PEPCK1 level peaks 24-48 h post-imbibition, and proteolytic forms of the protein are generated during this germination stage. Shorter PEPCK versions are also present in *Anana comosus* and *C. reinhardtii*, but in these cases, a transcriptional event leading to these shorted versions cannot be excluded (Martín *et al.* 2011; Torresi *et al.* 2023).

In plants, the abundance of the proteolyzed form is low compared to the non-proteolized form (Walker and Leegood 1995; Walker *et al*. 1995; Rojas *et al.* (2021b)). Some authors argue that low-stoichiometry PTMs may be physiologically irrelevant. Nevertheless, studies have shown that low-stoichiometry PTMs can reflect specific modifications occurring at a particular time and location (Prus *et al.* 2019). For instance, if a modification is specific to a group of cells, the modified protein will be diluted in a protein extract, leading to lower abundance (Orsburn *et al*. 2022). This may be the case of PEPCK, as proteolysis has been demonstrated to occur only in the cotyledons and embryonic axis of pea seedlings (Delgado-Alvarado *et al.* 2007).

Truncated mutants of *Ath*PEPCK1 on the putative cleavage sites of *Ath*MC9 (Δ19 and Δ101 mutants) generated protein forms with similar kinetic parameters and quaternary structure compared to the WT enzyme. However, the activation by Mal and inhibition by Glc6P were abolished in the Δ101 mutant (Rojas *et al.* 2021b). Proteolysis during germination may serve as a mechanism to regulate enzyme levels and, consequently, modulate the gluconeogenic flux. Furthermore, the shorter versions of *Ath*PEPCK1 may fulfil different roles during the transition of seedlings from an autotrophic to a heterotrophic state. Interestingly, the proteolysis of PEPCK cleaved the N-terminal portion of the protein, which has been predicted to contain an intrinsically disordered motif and a major phosphorylation site. Intrinsically disordered motifs evolved to have a disordered structure under physiological conditions (Peterson *et al.* 2017). These motifs exhibit rapid conformational fluctuations and are involved in signalling cascades, protein–protein interaction modules, allosteric regulations and auto-inhibitory domains, and are usual targets of PTMs (Wright and Dyson 2014).

#### Phosphorylation.

In the leaves of C_4_ and CAM plants, phosphorylation of PEPCK in its N-terminal domain has been observed during the dark period (Walker and Leegood 1995; Leegood and Walker 1996). This phosphorylation was recreated *in vitro* by PEPC kinase and cAMP-dependent protein kinase (Walker and Leegood 1995). In crude extracts from the fodder *Megathyrsus maximus*, lower PEPCK activity was detected during the night, suggesting an inhibitory effect of phosphorylation (Walker *et al.* 2002). Nevertheless, purification of PEPCK from day and night samples resulted in preparations with different substrate affinities but no differences in specific activity when measured using *in vivo* assay conditions that assess maximum activity (Walker and Chen 2002). Only the enzyme from night samples exhibited increased *K*_M_ for OAA and ATP. Therefore, PEPC and PEPCK are regulated through phosphorylation to prevent futile phosphorylation–dephosphorylation cycles (Leegood and Walker 2003; Bailey *et al.* 2007). During the day, in C_4_ and CAM plant leaves, dephosphorylation of both enzymes leads to PEPC inhibition and PEPCK activation. Conversely, during the night, when both enzymes are phosphorylated, the opposite activity conditions occur.

The recent development and optimization of mass spectrometry protocols led to unprecedented amounts of phosphoproteomic data (Smith and Kelleher 2013; Aebersold *et al.* 2018; Willems *et al.* 2019). This technological progress has also enabled the detection and analysis of low-stoichiometry PTMs that were previously considered irrelevant (Prus *et al.* 2019). *Ath*PEPCK1 and *Ath*PEPCK2 undergo phosphorylation at multiple sites. Phosphomimetic mutants at Ser-62 exhibited increased enzyme activity, while mutants at Thr-56 decreased activity. The phosphomimetic mutant at Thr-66 displayed no difference compared to the WT enzyme (Shen *et al.* 2017). This information further supports the notion that *Ath*PEPCK1 is intricately regulated in a complex manner by different signals mediated by effectors and PTMs.

In *Zea mays*, mass spectrometry profiling of PEPCK revealed that light conditions altered its phosphorylation status (Chao *et al.* 2014). In Arabidopsis, treatment with flg22, a 22-amino acid peptide derived from *Pseudomonas syringae* flagellin, resulted in increased phosphorylation of Thr-22 and decreased phosphorylation of Ser-62 and Thr-66 (Rayapuram *et al.* 2014, 2018). These findings suggest that phosphorylation may transduce different environmental signals. Although unequivocal evidence of the protein kinase(s) responsible for PEPCKs phosphorylation is lacking, studies have implicated SnRK2.2/SnRK2.3/SnRK2.6, MPK6, GSK3 and TOR (de la Fuente van Bentem *et al.* 2008; Wang *et al.* 2013; Rayapuram *et al.* 2018; van Leene *et al.* 2019).

### Open questions and future lines of research

Although significant research has been performed on PEPCKs’ function and regulation, many questions await answers: (i) How do different PTMs regulate enzyme activity, and how do they integrate with allosteric regulation? (ii) What are the biological outcomes of the different PTMs?; (iii) What are the modifying enzymes and signalling cascades acting on PEPCKs? (iv) Are dipeptides physiologically important modulators of PEPCK *in vivo*? and (v) Does PEPCK have an important role in non-PEPCK C4 and CAM plants, in particular, under stress situations? More research is needed to answer these questions but results will likely come from a combination of *in vitro* and *in vivo* biochemical approaches. The pioneer studies of Richard Leegood and Robert P. Walker with the enzyme purified from plant sources were key to studying the biochemical properties of plant PEPCKs (Leegood and Ap Rees 1978*a*, *b*; Leegood *et al.* 1996, 2003; Walker and Chen 2002; Walker and Leegood 1995; Walker *et al.* 1995, 1997, 2002). Nevertheless, having a recombinant system to produce high amounts of the enzyme and mutant versions will be important in searching for the biochemical effects of PTMs on the enzyme activity in detail.

In the era of Big Data in biology, vast amounts of transcriptomic, metabolomics and proteomic data are added each day to public databases. Some examples of useful tools are BRENDA (Chang *et al.* 2021), Plant PTM viewer (Willems *et al.* 2019), PhosPhat (Durek *et al.* 2010), BAR (Winter *et al.* 2007), and TAIR (Huala *et al.* 2001). This gives plant biochemists enormous potential to explore novel modes of metabolic regulation in combination with other biochemical techniques (Hartman *et al.* 2023). A pipeline to translate the biochemical knowledge to metabolic engineering research would start with pure preparations of the enzymes and mutant versions that mimic the PTMs to clearly understand their regulation. Enzymes could also be engineered to introduce new regulatory modes or abolish the existing ones (Erb *et al.* 2017). Then, the promising mutants could be expressed *in vivo*, followed by the analysis of the resulting plants’ phenotypic and metabolic effects. The PEPCK example is similar to what happens with other highly regulated enzymes (e.g. glyceraldehyde-3-phosphate dehydrogenases, ADP-glucose pyrophosphorylases, sucrose synthases, Glc6P dehydrogenases, among other interesting examples). The current challenge is integrating data from multiple regulations on enzyme activity, generated *in vitro* and *in vivo*, to develop general models and reduce the existing gap between both approaches (for some good examples, we suggest reading the work of Liebermeister *et al.* 2006; Blätke *et al.* 2019; Treves *et al.* 2022). This would lead to more biochemical knowledge and understanding that would certainly speed up agrobiotechnology innovations.

## Data Availability

No new data were generated in support of this research.

## Notes

**Phenome, Genome and Environment. Chief Editor:** Colleen Doherty

## References

[CIT0001] Ackoff RL. 1999. Ackoff’s best: his classic writings on management. New York: John Wiley & Sons.

[CIT0002] Aebersold R , Agar JN, Amster IJ, Baker MS, Bertozzi CR, Boja ES, Costello CE, Cravatt BF, Fenselau C, Garcia BA, et al. 2018. How many human proteoforms are there? Nature Chemical Biology 14:206–214. 29443976 10.1038/nchembio.2576 PMC5837046

[CIT0003] Aich S , Delbaere LTJ. 2007. Phylogenetic study of the evolution of PEP-carboxykinase. Evolutionary Bioinformatics 3:333–340. 19461981 PMC2684135

[CIT0004] Arrivault S , Obata T, Szecowka M, Mengin V, Guenther M, Hoehne M, Fernie AR, Stitt M. 2017. Metabolite pools and carbon flow during C_4_ photosynthesis in maize: ^13^CO_2_ labeling kinetics and cell type fractionation. Journal of Experimental Botany 68:283–298. 27834209 10.1093/jxb/erw414 PMC5853532

[CIT0005] Avin-Wittenberg T , Bajdzienko K, Wittenberg G, Alseekh S, Tohge T, Bock R, Giavalisco P, Fernie AR. 2015. Global analysis of the role of autophagy in cellular metabolism and energy homeostasis in Arabidopsis seedlings under carbon starvation. The Plant Cell 27:306–322. 25649436 10.1105/tpc.114.134205 PMC4456922

[CIT0006] Bailey KJ , Gray JE, Walker RP, Leegood RC. 2007. Coordinate regulation of phospho*enol*pyruvate carboxylase and phospho*enol*pyruvate carboxykinase by light and CO_2_ during C_4_ photosynthesis. Plant Physiology 144:479–486. 17337522 10.1104/pp.106.093013 PMC1913779

[CIT0007] Bassham DC. 2009. Function and regulation of macroautophagy in plants. Biochimica et Biophysica Acta, Molecular Cell Research 1793:1397–1403. 10.1016/j.bbamcr.2009.01.001 19272302

[CIT0008] Black CC , Chen JQ, Doong RL, et al. 1996. Alternative carbohydrate reserves used in the daily cycle of crassulacean acid metabolism. In: Winter K, Smith JAC, eds. Crassulacean acid metabolism. Ecological studies, Vol. 114. Berlin, Heidelberg: Springer, 31–45.

[CIT0009] Blätke MA , Bräutigam A. 2019. Evolution of C_4_ photosynthesis predicted by constraint-based modelling. eLife 8:1–24. 10.7554/eLife.49305 PMC6905489 31799932

[CIT0010] Borland AM , Técsi LI, Leegood RC, Walker RP. 1998. Inducibility of crassulacean acid metabolism (CAM) in *Clusia* species; physiological/biochemical characterisation and intercellular localization of carboxylation and decarboxylation processes in three species which exhibit different degrees of CAM. Planta 205:342–351.

[CIT0011] Brown NJ , Palmer BG, Stanley S, Hajaji H, Janacek SH, Astley HM, Parsley K, Kajala K, Quick WP, Trenkamp S, et al. 2010. C_4_ acid decarboxylases required for C_4_ photosynthesis are active in the mid-vein of the C_3_ species *Arabidopsis thaliana*, and are important in sugar and amino acid metabolism. The Plant Journal 61:122–133. 19807880 10.1111/j.1365-313X.2009.04040.x

[CIT0012] Burnell JN. 1986. Purification and properties of phospho*enol*pyruvate carboxykinase from C_4_ plants. Australian Journal of Plant Physiology 13:577–587.

[CIT0013] Cabello-Pasini A , Swift H, Smith GJ, Alberte RS. 2001. Phospho*enol*pyruvate carboxykinase from the marine diatom *Skeletonema costatum* and the phaeophyte *Laminaria setchellii*. II. Immunological characterization and subcellular localization. Botanica Marina 44:199–207.

[CIT0014] Cacefo V , Ribas AF, Zilliani RR, Neris DM, Domingues DS, Moro AL, Vieira LGE. 2019. Decarboxylation mechanisms of C_4_ photosynthesis in *Saccharum* spp.: increased PEPCK activity under water-limiting conditions. BMC Plant Biology 19:1–14. 30991938 10.1186/s12870-019-1745-7 PMC6469216

[CIT0015] Chang A , Jeske L, Ulbrich S, Hofmann J, Koblitz J, Schomburg I, Neumann-Schaal M, Jahn D, Schomburg D. 2021. BRENDA, the ELIXIR core data resource in 2021: new developments and updates. Nucleic Acids Research 49:D498–D508. 33211880 10.1093/nar/gkaa1025 PMC7779020

[CIT0016] Chao Q , Liu XY, Mei YC, Gao Z-F, Chen Y-B, Qian C-R, Hao Y-B, Wang B-C. 2014. Light-regulated phosphorylation of maize phospho*enol*pyruvate carboxykinase plays a vital role in its activity. Plant Molecular Biology 85:95–105. 24435212 10.1007/s11103-014-0171-3

[CIT0017] Chapman KSR , Hatch MD. 1981. Aspartate decarboxylation in bundle sheath cells of *Zea mays* and its possible contribution to C_3_ photosynthesis. Functional Plant Biology 8:237–248.

[CIT0018] Chapman KSR , Berry JA, Hatch MD. 1980. Photosynthetic metabolism in bundle sheath cells of the C_4_ species *Zea mays*: sources of ATP and NADPH and the contribution of photosystem II. Archives of Biochemistry and Biophysics 202:330–341. 7458323 10.1016/0003-9861(80)90435-x

[CIT0019] Chen Z-H , Walker RP, Acheson RM, Técsi LI, Wingler A, Lea PJ, Leegood RC. 2000. Are isocitrate lyase and phospho*enol*pyruvate carboxykinase involved in gluconeogenesis during senescence of barley leaves and cucumber cotyledons? Plant and Cell Physiology 41:960–967. 11038056 10.1093/pcp/pcd021

[CIT0020] Chen ZH , Walker RP, Acheson RM, Leegood RC. 2002. Phospho*enol*pyruvate carboxykinase assayed at physiological concentrations of metal ions has a high affinity for CO_2_. Plant Physiology 128:160–164. 11788761 PMC148961

[CIT0021] Chen ZH , Walker RP, Técsi LI, Lea PJ, Leegood RC. 2004. Phospho*enol*pyruvate carboxykinase in cucumber plants is increased both by ammonium and by acidification, and is present in the phloem. Planta 219:48–58. 14991407 10.1007/s00425-004-1220-y

[CIT0022] Chiba Y , Kamikawa R, Nakada-Tsukui K, Saito-Nakano Y, Nozaki T. 2015. Discovery of PPi-type phospho*enol*pyruvate carboxykinase genes in eukaryotes and bacteria. Journal of Biological Chemistry 290:23960–23970. 26269598 10.1074/jbc.M115.672907 PMC4583022

[CIT0023] Chiba Y , Miyakawa T, Shimane Y, Takai K, Tanokura M, Nozaki T. 2019. Structural comparisons of phospho*enol*pyruvate carboxykinases reveal the evolutionary trajectories of these phosphodiester energy conversion enzymes. Journal of Biological Chemistry 294:19269–19278. 31662435 10.1074/jbc.RA119.010920 PMC6916476

[CIT0024] Choi DS , Kim NH, Hwang BK. 2015. The pepper phospho*enol*pyruvate carboxykinase *Ca*PEPCK1 is involved in plant immunity against bacterial and oomycete pathogens. Plant Molecular Biology 89:99–111. 26233534 10.1007/s11103-015-0354-6

[CIT0025] Daloso DM , Medeiros DB, dos Anjos L, Yoshida T, Araújo WL, Fernie AR. 2017. Metabolism within the specialized guard cells of plants. New Phytologist 216:1018–1033. 28984366 10.1111/nph.14823

[CIT0026] Dammann O. 2019. Data, information, evidence, and knowledge: a proposal for health informatics and data science. Online Journal of Public Health Informatics 10:e224. 30931086 10.5210/ojphi.v10i3.9631 PMC6435353

[CIT0027] De La Fuente Van Bentem S , Anrather D, Dohnal I, Roitinger E, Csaszar E, Joore J, Buijnink J, Carreri A, Forzani C, Lorkovic ZJ, et al. 2008. Site-specific phosphorylation profiling of Arabidopsis proteins by mass spectrometry and peptide chip analysis. Journal of Proteome Research 7:2458–2470. 18433157 10.1021/pr8000173

[CIT0028] Delgado-Alvarado A , Walker RP, Leegood RC. 2007. Phospho*enol*pyruvate carboxykinase in developing pea seeds is associated with tissues involved in solute transport and is nitrogen-responsive. Plant, Cell and Environment 30:225–235. 10.1111/j.1365-3040.2006.01622.x 17238913

[CIT0029] Dittrich P , Campbell WH, Black CC. 1973. Phospho*enol*pyruvate carboxykinase in plants exhibiting crassulacean acid metabolism. Plant Physiology 52:357–361. 16658562 10.1104/pp.52.4.357 PMC366502

[CIT0030] Doppler M , Kluger B, Bueschl C, Steiner B, Buerstmayr H, Lemmens M, Krska R, Adam G, Schuhmacher R. 2019. Stable isotope-assisted plant metabolomics: investigation of phenylalanine-related metabolic response in wheat upon treatment with the fusarium virulence factor deoxynivalenol. Frontiers in Plant Science 10:1–18. 31736983 10.3389/fpls.2019.01137 PMC6831647

[CIT0031] Durek P , Schmidt R, Heazlewood JL, Jones A, MacLean D, Nagel A, Kersten B, Schulze WX. 2010. PhosPhAt: the *Arabidopsis thaliana* phosphorylation site database. An update. Nucleic Acids Research 38:D828–D834. 19880383 10.1093/nar/gkp810 PMC2808987

[CIT0032] Eastmond PJ , Astley HM, Parsley K, Aubry S, Williams BP, Menard GN, Craddock CP, Nunes-Nesi A, Fernie AR, Hibberd JM. 2015. Arabidopsis uses two gluconeogenic gateways for organic acids to fuel seedling establishment. Nature Communications 6:6659. 10.1038/ncomms7659 PMC4403315 25858700

[CIT0033] Erb TJ , Jones PR, Bar-Even A. 2017. Synthetic metabolism: metabolic engineering meets enzyme design. Current Opinion in Chemical Biology 37:56–62. 28152442 10.1016/j.cbpa.2016.12.023 PMC7610756

[CIT0034] Escamez S , Andre D, Zhang B, Bollhöner B, Pesquet E, Tuominen H. 2016. METACASPASE9 modulates autophagy to confine cell death to the target cells during Arabidopsis vascular xylem differentiation. Biology Open 5:122–129. 26740571 10.1242/bio.015529 PMC4823987

[CIT0035] Escamez S , Stael S, Vainonen JP, Willems P, Jin H, Kimura S, van Breusegem F, Gevaert K, Wrzaczek M, Tuominen H. 2019. Extracellular peptide Kratos restricts cell death during vascular development and stress in Arabidopsis. Journal of Experimental Botany 70:2199–2210. 30753577 10.1093/jxb/erz021 PMC6460963

[CIT0036] Famiani F , Moscatello S, Ferradini N, Gardi T, Battistelli A, Walker RP. 2014. Occurrence of a number of enzymes involved in either gluconeogenesis or other processes in the pericarp of three cultivars of grape (*Vitis vinifera L.*) during development. Plant Physiology and Biochemistry 84:261–270. 25306529 10.1016/j.plaphy.2014.10.003

[CIT0037] Famiani F , Farinelli D, Frioni T, Palliotti A, Battistelli A, Moscatello S, Walker RP. 2016. Malate as substrate for catabolism and gluconeogenesis during ripening in the pericarp of different grape cultivars. Biologia Plantarum 60:155–162.

[CIT0038] Fukuda W , Fukui T, Atomi H, Imanaka T. 2004. First characterization of an archaeal GTP-dependent phospho*enol*pyruvate carboxykinase from the hyperthermophilic archaeon *Thermococcus kodakaraensis* KOD1. Journal of Bacteriology 186:4620–4627. 15231795 10.1128/JB.186.14.4620-4627.2004 PMC438638

[CIT0039] Furbank RT. 2011. Evolution of the C_4_ photosynthetic mechanism: are there really three C_4_ acid decarboxylation types? Journal of Experimental Botany 62:3103–3108. 21511901 10.1093/jxb/err080

[CIT0040] Furumoto T , Hata S, Izui K. 1999. cDNA cloning and characterization of maize phospho*enol*pyruvate carboxykinase, a bundle sheath cell-specific enzyme. Plant Molecular Biology 41:301–311. 10598098 10.1023/a:1006317120460

[CIT0041] Giordano M , Beardall J, Raven JA. 2005. CO_2_ concentrating mechanisms in algae: mechanisms, environmental modulation, and evolution. Annual Review of Plant Biology 56:99–131. 10.1146/annurev.arplant.56.032604.144052 15862091

[CIT0042] Hatch MD. 1971. The C_4_-pathway of photosynthesis. Evidence for an intermediate pool of carbon dioxide and the identity of the donor C_4_-dicarboxylic acid. Biochemical Journal 125:425–432. 5144745 10.1042/bj1250425 PMC1178076

[CIT0043] Hatch MD , Mau S. 1977. Properties of phospho*enol*pyruvate carboxykinase operative in C_4_ pathway photosynthesis. Functional Plant Biology 4:207–216.

[CIT0044] Hatch MD , Kagawa T, Craig S. 1975. Subdivision of C_4_-pathway species based on differing C_4_ acid decarboxylating systems and ultrastructural features. Functional Plant Biology 2:111–128.

[CIT0145] Hartman MD , Rojas BE, Iglesias AA, Figueroa CM. The involvement of allosteric effectors and post‐translational modifications in the control of plant central carbon metabolism. The Plant Journal 2023;114:1037–1058. 37092344 10.1111/tpj.16215

[CIT0045] Henninger M , Pedrotti L, Krischke M, Draken J, Wildenhain T, Fekete A, Rolland F, Müller MJ, Fröschel C, Weiste C, et al. 2022. The evolutionarily conserved kinase SnRK1 orchestrates resource mobilization during Arabidopsis seedling establishment. The Plant Cell 34:616–632. 34755865 10.1093/plcell/koab270 PMC8774017

[CIT0046] Herman EM , Larkins BA. 1999. Protein storage bodies and vacuoles. Plant Cell 11:601–614. 10213781 10.1105/tpc.11.4.601 PMC144198

[CIT0047] Holtum J , Osmond C. 1981. The gluconeogenic metabolism of pyruvate during deacidification in plants with crassulacean acid metabolism. Functional Plant Biology 8:31–44.

[CIT0048] Holyoak T , Sullivan SM, Nowak T. 2006. Structural insights into the mechanism of PEPCK catalysis. Biochemistry 45:8254–8263. 16819824 10.1021/bi060269g

[CIT0049] Huala E , Dickerman A, Garcia-Hernandez M, et al. 2001. The Arabidopsis Information Resource (TAIR): a comprehensive database and web-based information retrieval, analysis, and visualization system for a model plant. Nucleic Acids Research 29:102–105. 11125061 10.1093/nar/29.1.102 PMC29827

[CIT0050] Huang YX , Goto Y, Nonaka S, Fukuda N, Ezura H, Matsukura C. 2015a. Overexpression of the phospho*enol*pyruvate carboxykinase gene (*Sl*PEPCK) promotes soluble sugar accumulation in fruit and post-germination growth of tomato (*Solanum lycopersicum* L.). Plant Biotechnology 32:281–289.

[CIT0051] Huang YX , Yin YG, Sanuki A, Fukuda N, Ezura H, Matsukura C. 2015b. Phospho*enol*pyruvate carboxykinase (PEPCK) deficiency affects the germination, growth and fruit sugar content in tomato (*Solanum lycopersicum* L.). Plant Physiology and Biochemistry 96:417–425. 26381194 10.1016/j.plaphy.2015.08.021

[CIT0052] Ito J , Batth TS, Petzold CJ, Redding-Johanson AM, Mukhopadhyay A, Verboom R, Meyer EH, Millar AH, Heazlewood JL. 2011. Analysis of the Arabidopsis cytosolic proteome highlights subcellular partitioning of central plant metabolism. Journal of Proteome Research 10:1571–1582. 21166475 10.1021/pr1009433

[CIT0053] John CR , Smith-Unna RD, Woodfield H, Covshoff S, Hibberd JM. 2014. Evolutionary convergence of cell-specific gene expression in independent lineages of C_4_ grasses. Plant Physiology 165:62–75. 24676859 10.1104/pp.114.238667 PMC4012605

[CIT0054] Johnson TA , McLeod MJ, Holyoak T. 2016. Utilization of substrate intrinsic binding energy for conformational change and catalytic function in phospho*enol*pyruvate carboxykinase. Biochemistry 55:575–587. 26709450 10.1021/acs.biochem.5b01215

[CIT0055] Kenny AJ , Booth AG, George SG, Ingram J, Kershaw D, Wood EJ, Young AR. 1976. Dipeptidyl peptidase IV, a kidney brush border serine peptidase. Biochemical Journal 157:169–182. 962853 10.1042/bj1570169 PMC1163828

[CIT0056] Kim DJ , Smith SM. 1994. Molecular cloning of cucumber phospho*enol*pyruvate carboxykinase and developmental regulation of gene expression. Plant Molecular Biology 26:423–434. 7948888 10.1007/BF00039551

[CIT0057] Kim SM , Bae C, Oh SK, Choi D. 2013. A pepper (*Capsicum annuum* L.) metacaspase 9 (*camc9*) plays a role in pathogen-induced cell death in plants. Molecular Plant Pathology 14:557–566. 23522353 10.1111/mpp.12027 PMC6638822

[CIT0058] Kwon SI , Hwang DJ. 2013. Expression analysis of the metacaspase gene family in Arabidopsis. Journal of Plant Biology 56:391–398.

[CIT0059] Larson LA , Beevers H. 1965. Amino acid metabolism in young pea seedlings. Plant Physiology 40:424–432. 16656105 10.1104/pp.40.3.424 PMC550310

[CIT0060] Lea PJ , Chen Z-H, Leegood RC, Walker RP. 2001. Does phospho*enol*pyruvate carboxykinase have a role in both amino acid and carbohydrate metabolism? Amino Acids 20:225–241. 11354601 10.1007/s007260170041

[CIT0061] Lee MH , Hebda CA, Nowak T. 1981. The role of cations in avian liver phospho*enol*pyruvate carboxykinase catalysis: activation and regulation. Journal of Biological Chemistry 256:12793–12801. 6796577

[CIT0062] Leegood RC , Ap Rees T. 1978a. Phosphoenolpyruvate carboxykinase and gluconeogenesis in cotyledons of *Cucurbita pepo*. Biochimica et Biophysica Acta, Enzymology 524:207–218. 10.1016/0005-2744(78)90119-5 656445

[CIT0063] Leegood RC , Ap Rees T. 1978b. Identification of the regulatory steps in gluconeogenesis in cotyledons of *Cucurbita pepo*. BBA—General Subjects 542:1–11. 208645 10.1016/0304-4165(78)90226-x

[CIT0064] Leegood RC , Walker RP. 1996. Phosphorylation of phospho*enol*pyruvate carboxykinase in plants. Studies in plants with C_4_ photosynthesis and crassulacean acid metabolism and in germinating seeds. Biochemical Journal 317:653–658. 8760346 10.1042/bj3170653 PMC1217536

[CIT0065] Leegood RC , Walker RP. 2003. Regulation and roles of phospho*enol*pyruvate carboxykinase in plants. Archives of Biochemistry and Biophysics 414:204–210. 12781772 10.1016/s0003-9861(03)00093-6

[CIT0066] Liebermeister W , Klipp E. 2006. Bringing metabolic networks to life: integration of kinetic, metabolic, and proteomic data. Theoretical Biology and Medical Modelling 3:42. 17173670 10.1186/1742-4682-3-42 PMC1781439

[CIT0067] Lim SD , Lee S, Choi WG, Yim WC, Cushman JC. 2019. Laying the foundation for crassulacean acid metabolism (CAM) biodesign: expression of the C_4_ metabolism cycle genes of CAM in Arabidopsis. Frontiers in Plant Science 10:101. 30804970 10.3389/fpls.2019.00101 PMC6378705

[CIT0068] Lohaus G , Burba M, Heldt HW. 1994. Comparison of the contents of sucrose and amino acids in the leaves, phloem sap and taproots of high and low sugar-producing hybrids of sugar beet (*Beta vulgaris* L.). Journal of Experimental Botany 45:1097–1101.

[CIT0069] Maier A , Zell MB, Maurino VG. 2011. Malate decarboxylases: evolution and roles of NAD(P)-ME isoforms in species performing C_4_ and C_3_ photosynthesis. Journal of Experimental Botany 62:3061–3069. 21459769 10.1093/jxb/err024

[CIT0070] Majeran W , Friso G, Ponnal L, et al. 2010. Structural and metabolic transitions of C_4_ leaf development and differentiation defined by microscopy and quantitative proteomics in maize. The Plant Cell 22:3509–3542. 21081695 10.1105/tpc.110.079764 PMC3015116

[CIT0071] Malone S , Chen Z-H, Bahrami AR, Walker RP, Gray JE, Leegood RC. 2007. Phospho*enol*pyruvate carboxykinase in Arabidopsis: changes in gene expression, protein and activity during vegetative and reproductive development. Plant and Cell Physiology 48:441–450. 17283014 10.1093/pcp/pcm014

[CIT0072] Marquardt A , Henry RJ, Botha FC. 2021. Effect of sugar feedback regulation on major genes and proteins of photosynthesis in sugarcane leaves. Plant Physiology and Biochemistry 158:321–333. 33250321 10.1016/j.plaphy.2020.11.022

[CIT0073] Martín M , Plaxton WC, Podestá FE. 2007. Activity and concentration of non-proteolyzed phospho*enol*pyruvate carboxykinase in the endosperm of germinating castor oil seeds: effects of anoxia on its activity. Physiologia Plantarum 130:484–494.

[CIT0074] Martín M , Rius SP, Podestá FE. 2011. Two phospho*enol*pyruvate carboxykinases coexist in the crassulacean acid metabolism plant *Ananas comosus*. Isolation and characterization of the smaller 65kDa form. Plant Physiology and Biochemistry 49:646–653. 21398135 10.1016/j.plaphy.2011.02.015

[CIT0075] Matte A , Tari LW, Goldie H, Delbaere LTJ. 1997. Structure and mechanism of phospho*enol*pyruvate carboxykinase. Journal of Biological Chemistry 272:8105–8108. 9139042 10.1074/jbc.272.13.8105

[CIT0076] Meister M , Agostino A, Hatch MD. 1996. The roles of malate and aspartate in C_4_ photosynthetic metabolism of *Flaveria bidentis* (L.). Planta 199:262–269.

[CIT0077] Moreno JC , Rojas BE, Vicente R, Gorka M, Matz T, Chodasiewicz M, Peralta-Ariza JS, Zhang Y, Alseekh S, Childs D, et al. 2021. Tyr-Asp inhibition of glyceraldehyde 3-phosphate dehydrogenase affects plant redox metabolism. The EMBO Journal 40:1–16. 10.15252/embj.2020106800 PMC8327957 34156108

[CIT0078] Muhaidat R , McKown AD. 2013. Significant involvement of PEPCK in carbon assimilation of C_4_ eudicots. Annals of Botany 111:577–589. 23388881 10.1093/aob/mct017 PMC3605952

[CIT0079] Mukundan NS , Banerjee S, Kumar S, Satyamoorthy K, Babu VS. 2023. C_4_ equivalent decarboxylation competence in tropical orchids. Journal of Plant Biology 66:163–180.

[CIT0080] O´Leary M. 1992. Catalytic strategies in enzymic carboxylation and decarboxylation. The Enzymes 20:235–269.

[CIT0081] Orsburn BC , Yuan Y, Bumpus NN. 2022. Insights into protein post-translational modification landscapes of individual human cells by trapped ion mobility time-of-flight mass spectrometry. Nature Communications 13:7246. 10.1038/s41467-022-34919-w PMC9700839 36433961

[CIT0082] Osorio S , Vallarino JG, Szecowka M, Ufaz S, Tzin V, Angelovici R, Galili G, Fernie AR. 2013. Alteration of the interconversion of pyruvate and malate in the plastid or cytosol of ripening tomato fruit invokes diverse consequences on sugar but similar effects on cellular organic acid, metabolism, and transitory starch accumulation. Plant Physiology 161:628–643. 23250627 10.1104/pp.112.211094 PMC3561009

[CIT0083] Owen OE , Kalhan SC, Hanson RW. 2002. The key role of anaplerosis and cataplerosis for citric acid cycle function. Journal of Biological Chemistry 277:30409–30412. 12087111 10.1074/jbc.R200006200

[CIT0084] Peckmann K , Willert DJ, Von Martin CE, et al. 2012. Mitochondrial respiration in ME-CAM, PEPCK-CAM, and C_3_ succulents: ­comparative operation of the cytochrome, alternative, and rotenone-resistant pathways. Journal of Experimental Botany 63:2909–2919. 22330897 10.1093/jxb/err458

[CIT0085] Penfield S , Rylott EL, Gilday AD, Graham S, Larson TR, Graham IA. 2004. Reserve mobilization in the Arabidopsis endosperm fuels hypocotyl elongation in the dark, is independent of abscisic acid, and requires *phosphoenolpyruvate carboxykinase1*. The Plant Cell 16:2705–2718. 15367715 10.1105/tpc.104.024711 PMC520966

[CIT0086] Penfield S , Clements S, Bailey KJ, Gilday AD, Leegood RC, Gray JE, Graham IA. 2012. Expression and manipulation of *PHOSPHOENOLPYRUVATE CARBOXYKINASE 1* identifies a role for malate metabolism in stomatal closure. The Plant Journal 69:679–688. 22007864 10.1111/j.1365-313X.2011.04822.x

[CIT0087] Peterson LX , Roy A, Christoffer C, Terashi G, Kihara D. 2017. Modeling disordered protein interactions from biophysical principles. PLoS Computational Biology 13:e1005485. 28394890 10.1371/journal.pcbi.1005485 PMC5402988

[CIT0088] Pick TR , Brautigam A, Schluter U, Denton AK, Colmsee C, Scholz U, Fahnenstich H, Pieruschka R, Rascher U, Sonnewald U, et al. 2011. Systems analysis of a maize leaf developmental gradient redefines the current C_4_ model and provides candidates for regulation. The Plant Cell 23:4208–4220. 22186372 10.1105/tpc.111.090324 PMC3269860

[CIT0089] Pittman JK. 2005. Managing the manganese: molecular mechanisms of manganese transport and homeostasis. New Phytologist 167:733–742. 16101910 10.1111/j.1469-8137.2005.01453.x

[CIT0090] Portis AR. 2001. Photosynthetic carbon metabolism. In: Encyclopedia of Life Sciences, eds. New Jersey: John Wiley & Sons, Ltd, 1–6.

[CIT0091] Prus G , Hoegl A, Weinert BT, Choudhary C. 2019. Analysis and interpretation of protein post-translational modification site stoichiometry. Trends in Biochemical Sciences 44:943–960. 31296352 10.1016/j.tibs.2019.06.003

[CIT0092] Quiquampoix H , Loughman BC, Ratcliffe RG. 1993. A ^31^P-NMR study of the uptake and compartmentation of manganese by maize roots. Journal of Experimental Botany 44:1819–1827.

[CIT0093] Raineri J , Hartman MD, Chan RL, Iglesias AA, Ribichich KF. 2016. A sunflower WRKY transcription factor stimulates the mobilization of seed-stored reserves during germination and post-germination growth. Plant Cell Reports 35:1875–1890. 27251125 10.1007/s00299-016-2002-2

[CIT0094] Rayapuram N , Bonhomme L, Bigeard J, Haddadou K, Przybylski C, Hirt H, Pflieger D. 2014. Identification of novel PAMP-triggered phosphorylation and dephosphorylation events in *Arabidopsis thaliana* by quantitative phosphoproteomic analysis. Journal of Proteome Research 13:2137–2151. 24601666 10.1021/pr401268v

[CIT0095] Rayapuram N , Bigeard J, Alhoraibi H, Bonhomme L, Hesse A-M, Vinh J, Hirt H, Pflieger D. 2018. Quantitative phosphoproteomic analysis reveals shared and specific targets of Arabidopsis mitogen-activated protein kinases (MAPKs) MPK3, MPK4, and MPK6. Molecular and Cellular Proteomics 17:61–80. 29167316 10.1074/mcp.RA117.000135 PMC5750851

[CIT0096] Reinfelder JR , Milligan AJ, Morel FMM. 2004. The role of the C_4_ pathway in carbon accumulation and fixation in a marine diatom. Plant Physiology 135:2106–2111. 15286292 10.1104/pp.104.041319 PMC520782

[CIT0097] Reiskind JB , Bowes G. 1991. The role of phospho*enol*pyruvate carboxykinase in a marine macroalga with C_4_-like photosynthetic characteristics. Proceedings of the National Academy of Sciences of the United States of America 88:2883–2887. 11607173 10.1073/pnas.88.7.2883 PMC51344

[CIT0098] Robaina-Estévez S , Daloso DM, Zhang Y, Fernie AR, Nikoloski Z. 2017. Resolving the central metabolism of Arabidopsis guard cells. Scientific Reports 7:1–13. 28814793 10.1038/s41598-017-07132-9 PMC5559522

[CIT0099] Rojas BE , Hartman MD, Figueroa CM, Leaden L, Podestá FE, Iglesias AA. 2019. Biochemical characterization of phospho*enol*pyruvate carboxykinases from *Arabidopsis thaliana*. Biochemical Journal 476:2939–2952. 31548269 10.1042/BCJ20190523

[CIT0100] Rojas BE , Figueroa CM, Iglesias AA. 2021a. Carbon assimilation and partitioning in crop plants: a biochemical and physiological view. In: Pessarakli M, ed. Handbook of plant and crop physiology. Florida: CRC Press, Chapter 22.

[CIT0101] Rojas BE , Hartman MD, Figueroa CM, Iglesias AA. 2021b. Proteolytic cleavage of *Arabidopsis thaliana* phospho*enol*pyruvate carboxykinase-1 modifies its allosteric regulation. Journal of Experimental Botany 72:2514–2524. 33315117 10.1093/jxb/eraa583

[CIT0102] Rylott EL , Hooks MA, Graham IA. 2001. Co-ordinate regulation of genes involved in storage lipid mobilization in *Arabidopsis thaliana*. Biochemical Society Transactions 29:283–287. 11356168 10.1042/0300-5127:0290283

[CIT0103] Rylott EL , Gilday AD, Graham IA. 2003. The gluconeogenic enzyme phospho*enol*pyruvate carboxykinase in Arabidopsis is essential for seedling establishment. Plant Physiology 131:1834–1842. 12692343 10.1104/pp.102.019174 PMC166940

[CIT0104] Saez-Vasquez J , Raynal M, Delseny M. 1995. A rapeseed cold-inducible transcript encodes a phospho*enol*pyruvate carboxykinase. Plant Physiology 109:611–618. 7480349 10.1104/pp.109.2.611 PMC157627

[CIT0105] Sage RF. 2004. The evolution of C_4_ photosynthesis. New Phytologist 161:341–370. 33873498 10.1111/j.1469-8137.2004.00974.x

[CIT0106] Sage RF , Peixoto MM, Sage TL. 2013. Photosynthesis in sugarcane. In Botha PH and Moore FC, eds. Sugarcane: physiology, biochemistry, and functional biology. New Jersey: John Wiley & Sons, 121–154.

[CIT0107] Saito T , Matsukura C, Ban Y, Shoji K, Sugiyama M, Fukuda N, Nishimura S. 2008. Salinity stress affects assimilate metabolism at the gene-expression level during fruit development and improves fruit quality in tomato (*Solanum lycopersicum* L.). Journal of the Japanese Society for Horticultural Science 77:61–68.

[CIT0108] Sales CRG , Ribeiro RV, Hayashi AH, et al. 2018. Flexibility of C_4_ decarboxylation and photosynthetic plasticity in sugarcane plants under shading. Environmental and Experimental Botany 149:34–42.

[CIT0109] Shen Z , Dong XM, Gao ZF, Chao Q, Wang B-C. 2017. Phylogenic and phosphorylation regulation difference of phospho*enol*pyruvate carboxykinase of C_3_ and C_4_ plants. Journal of Plant Physiology 213:16–22. 28285130 10.1016/j.jplph.2017.02.008

[CIT0110] Shen W , Liu J, Li JF. 2019. Type-II metacaspases mediate the processing of plant elicitor peptides in Arabidopsis. Molecular Plant 12:1524–1533. 31454707 10.1016/j.molp.2019.08.003

[CIT0111] Siu PML , Wood HG, Stjernholm RL. 1961. Fixation of CO_2_ by phospho*enol*pyruvic carboxytransphosphorylase. Journal of Biological Chemistry 236:21–22.

[CIT0112] Smith LM , Kelleher NL; Consortium for Top Down Proteomics. 2013. Proteoform: a single term describing protein complexity. Nature Methods 10:186–187. 23443629 10.1038/nmeth.2369 PMC4114032

[CIT0113] Stewart CR , Beevers H. 1967. Gluconeogenesis from amino acids in germinating castor bean endosperm and its role in transport to the embryo. Plant Physiology 42:1587–1595. 16656694 10.1104/pp.42.11.1587 PMC1086768

[CIT0114] Stitt M , Gibon Y. 2014. Why measure enzyme activities in the era of systems biology? Trends in Plant Science 19:256–265. 24332227 10.1016/j.tplants.2013.11.003

[CIT0115] Strehmel N , Hoehenwarter W, Mönchgesang S, Majovsky P, Krüger S, Scheel D, Lee J. 2017. Stress-related mitogen-activated protein kinases stimulate the accumulation of small molecules and proteins in *Arabidopsis thaliana* root exudates. Frontiers in Plant Science 8:1–13. 28785276 10.3389/fpls.2017.01292 PMC5520323

[CIT0116] Sweetman C , Deluc LG, Cramer GR, Ford CM, Soole KL. 2009. Regulation of malate metabolism in grape berry and other developing fruits. Phytochemistry 70:1329–1344. 19762054 10.1016/j.phytochem.2009.08.006

[CIT0117] Thirumalaikumar VP , Wagner M, Balazadeh S, et al. 2021. Autophagy is responsible for the accumulation of proteogenic dipeptides in response to heat stress in *Arabidopsis thaliana*. FEBS Journal 288:281–292. 32301545 10.1111/febs.15336

[CIT0118] Torresi F , Rodriguez FM, Gomez-Casati DF, Martín M. 2023. Two phospho*enol*pyruvate carboxykinases with differing biochemical properties in *Chlamydomonas reinhardtii*. FEBS Letters 597:585–597. 36708098 10.1002/1873-3468.14590

[CIT0119] Treves H , Küken A, Arrivault S, Ishihara H, Hoppe I, Erban A, Höhne M, Moraes TA, Kopka J, Szymanski J, et al. 2022. Carbon flux through photosynthesis and central carbon metabolism show distinct patterns between algae, C_3_ and C_4_ plants. Nature Plants 8:78–91. 34949804 10.1038/s41477-021-01042-5 PMC8786664

[CIT0120] Trípodi KEJ , Rojas BE, Iglesias AA, et al. 2021. CAM plants as crops: metabolically flexible, hardy plants for a changing world. In: Pessarakli M, ed. Handbook of plant and crop physiology, Chapter 48. Boca Raton, FL: CRC Press, 1083–1098.

[CIT0121] Tsiatsiani L , Timmerman E, Bock PD, et al. 2013. The Arabidopsis METACASPASE9 degradome. The Plant Cell 25:2831–2847. 23964026 10.1105/tpc.113.115287 PMC3784583

[CIT0122] van Leene J , Han C, Gadeyne A, Eeckhout D, Matthijs C, Cannoot B, de Winne N, Persiau G, van de Slijke E, van de Cotte B, et al. 2019. Capturing the phosphorylation and protein interaction landscape of the plant TOR kinase. Nature Plants 5:316–327. 30833711 10.1038/s41477-019-0378-z

[CIT0123] Vercammen D , Belenghi B, van de Cotte B, Beunens T, Gavigan J-A, de Rycke R, Brackenier A, Inzé D, Harris JL, VAN Breusegem F. 2006. Serpin 1 of *Arabidopsis thaliana* is a suicide inhibitor for metacaspase 9. Journal of Molecular Biology 364:625–636. 17028019 10.1016/j.jmb.2006.09.010

[CIT0124] Veyel D , Kierszniowska S, Kosmacz M, Sokolowska EM, Michaelis A, Luzarowski M, Szlachetko J, Willmitzer L, Skirycz A. 2017. System-wide detection of protein-small molecule complexes suggests extensive metabolite regulation in plants. Scientific Reports 7:1–8. 28205532 10.1038/srep42387 PMC5304321

[CIT0125] Veyel D , Sokolowska EM, Moreno JC, Kierszniowska S, Cichon J, Wojciechowska I, Luzarowski M, Kosmacz M, Szlachetko J, Gorka M, et al. 2018. PROMIS, global analysis of PROtein-metabolite interactions using size separation in *Arabidopsis thaliana*. Journal of Biological Chemistry 293:12440–12453. 29853640 10.1074/jbc.RA118.003351 PMC6093232

[CIT0126] Walker RP , Chen ZH. 2002. Phospho*enol*pyruvate carboxykinase: structure, function, and regulation. Advances in Botanical Research 38:93–189.

[CIT0127] Walker RP , Leegood RC. 1995. Purification, and phosphorylation *in vivo* and *in vitro*, of phospho*enol*pyruvate carboxykinase from cucumber cotyledons. FEBS Letters 362:70–74. 7698356 10.1016/0014-5793(95)00212-r

[CIT0128] Walker RP , Trevanion SJ, Leegood RC. 1995. Phospho*enol*pyruvate carboxykinase from higher plants: purification from cucumber and evidence of rapid proteolytic cleavage in extracts from a range of plant tissues. Planta 196:58–63.

[CIT0129] Walker RP , Acheson RM, Tecsi LI, Leegood RC. 1997. Phospho*enol*pyruvate carboxykinase in C_4_ plants: its role and regulation. Australian Journal of Plant Physiology 24:459–468.

[CIT0130] Walker RP , Chen ZH, Técsi LI, Famiani F, Lea PJ, Leegood RC. 1999. Phospho*enol*pyruvate carboxykinase plays a role in interactions of carbon and nitrogen metabolism during grape seed development. Planta 210:9–18. 10592027 10.1007/s004250050648

[CIT0131] Walker RP , Chen Z, Acheson RM, Leegood RC. 2002. Effects of phosphorylation on phospho*enol*pyruvate carboxykinase from the C_4_ plant Guinea Grass. Plant Physiology 128:165–172. 11788762 PMC148964

[CIT0132] Walker RP , Paoletti A, Leegood RC, Famiani F. 2016. Phosphorylation of phospho*enol*pyruvate carboxykinase (PEPCK) and phospho*enol*pyruvate carboxylase (PEPC) in the flesh of fruits. Plant Physiology and Biochemistry 108:323–327. 27497301 10.1016/j.plaphy.2016.07.021

[CIT0133] Walker RP , Chen ZH, Famiani F. 2021. Gluconeogenesis in plants: a key interface between organic acid/amino acid/lipid and sugar metabolism. Molecules 26:5129. 34500562 10.3390/molecules26175129 PMC8434439

[CIT0134] Wan L , Ross ARS, Yang J, Hegedus D D, Kermode A R. 2007. Phosphorylation of the 12S globulin cruciferin in wild-type and *abi1-1* mutant *Arabidopsis thaliana* (thale cress) seeds. Biochemical Journal 404:247–256. 17313365 10.1042/BJ20061569 PMC1868800

[CIT0135] Wang P , Xue L, Batelli G, Lee S, Hou Y-J, Van Oosten MJ, Zhang H, Tao WA, Zhu J-K. 2013. Quantitative phosphoproteomics identifies SnRK2 protein kinase substrates and reveals the effectors of abscisic acid action. Proceedings of the National Academy of Sciences of the United States of America 110:11205–11210. 23776212 10.1073/pnas.1308974110 PMC3703982

[CIT0136] Wang Y , Bräutigam A, Weber APM, Zhu X-G. 2014. Three distinct biochemical subtypes of C_4_ photosynthesis? A modelling analysis. Journal of Experimental Botany 65:3567–3578. 24609651 10.1093/jxb/eru058 PMC4085956

[CIT0137] Washburn JD , Strable J, Dickinson P, Kothapalli SS, Brose JM, Covshoff S, Conant GC, Hibberd JM, Pires JC. 2021. Distinct C_4_ sub-types and C_3_ bundle sheath isolation in the *Paniceae* grasses. Plant Direct 5:1–14. 10.1002/pld3.373 PMC8711749 34988355

[CIT0138] Weissmann S , Ma F, Furuyama K, Gierse J, Berg H, Shao Y, Taniguchi M, Allen DK, Brutnell TP. 2015. Interactions of C_4_ subtype metabolic activities and transport in maize are revealed through the characterization of DCT2 mutants. Plant Cell 28:466–484. 10.1105/tpc.15.00497 PMC4790864 26813621

[CIT0139] Willems P , Horne A, Van Parys T, Goormachtig S, De Smet I, Botzki A, Van Breusegem F, Gevaert K. 2019. The plant PTM viewer, a central resource for exploring plant protein modifications. The Plant Journal 99:752–762. 31004550 10.1111/tpj.14345

[CIT0140] Wingler A , Walker RP, Chen ZH, Leegood RC. 1999. Phospho*enol*pyruvate carboxykinase is involved in the decarboxylation of aspartate in the bundle sheath of maize. Plant Physiology 120:539–546. 10364405 10.1104/pp.120.2.539 PMC59292

[CIT0141] Winter D , Vinegar B, Ammar R, et al. 2007. An ‘electronic fluorescent pictograph’ browser for exploring and analyzing large-scale biological datasets. PLoS One 2:e718. 17684564 10.1371/journal.pone.0000718 PMC1934936

[CIT0142] Wright PE , Dyson JH. 2014. Intrinsically disordered proteins in cellular signaling and regulation. Nature Reviews Molecular Cell Biology 16:18–29. 10.1038/nrm3920 PMC4405151 25531225

[CIT0143] Wurtzel ET , Vickers CE, Hanson AD, Millar AH, Cooper M, Voss-Fels KP, Nikel PI, Erb TJ. 2019. Revolutionizing agriculture with synthetic biology. Nature Plants 5:1207–1210. 31740769 10.1038/s41477-019-0539-0

[CIT0144] Yang J , Pan Y, Bowler C, Zhang L, Hu H. 2016. Knockdown of phospho*enol*pyruvate carboxykinase increases carbon flux to lipid synthesis in *Phaeodactylum tricornutum*. Algal Research 15:50–58.

